# Research on Processing-Induced Chemical Variations in Polygonatum Cyrtonema Rhizome by Integrating Metabolomics and Glycomics

**DOI:** 10.3390/molecules27185869

**Published:** 2022-09-10

**Authors:** Tong Jiang, Tong Wu, Peiyun Gao, Lixia Wang, Xiaoyun Yang, Xiaoxu Chen, Yingying Chen, Chunyu Yue, Keqing Liang, Liying Tang, Zhuju Wang

**Affiliations:** 1 Institute of Chinese Materia Medica, China Academy of Chinese Medical Science, Beijing 100700, China; 2 College of Pharmacy, Henan University of Chinese Medicine, Zhengzhou 450046, China

**Keywords:** polygonatum cyrtonema rhizome, processing stage, processing chemistry, metabolomics, glycomics, overall chemical profiling

## Abstract

Polygonatum cyrtonema rhizome (PCR), the dried sweet rhizome of *Polygonatum cyrtonema* Hua, is commonly used as a tonic remedy and a functional food in Asia, Europe, and North America. Multiple components, including secondary metabolites, monosaccharides, oligosaccharides, and polysaccharides, collectively contribute to the therapeutic effects of PCR. Processing time exerts a significant influence on the quality of PCR, but the various processing stages have not been comprehensively chemically profiled. It is urgent to study processing-induced chemical variations in PCR to control the processing degree. In this study, multiple chromatographic and mass spectrometric techniques were used in combination with multivariate statistical analysis to perform qualitative and quantitative research on secondary metabolites and carbohydrates in PCR during processing. The results demonstrated that PCR processing can be divided into three stages, namely the raw stage (0 h), the middle stage (1–6 h), and the late stage (8–18 h). Twenty differential compounds were screened from secondary metabolites and oligosaccharides to distinguish PCR in different processing stages. Furthermore, the chemical variations of Polygonatum cyrtonema polysaccharides (PCP) also entered a new stage after processing for 6 h. Multiple chemical mechanisms, including hydrolysis, oxidative decomposition, dehydration, Maillard reaction, and polymerization were involved in the processing. This work provides a scientific basis to reveal the relationship between processing stage and chemical variations.

## 1. Introduction

Polygonatum cyrtonema rhizome (PCR), called “*Huangjing*” in China, is a famous traditional medicine obtained from the rhizome of *Polygonatum cyrtonema* Hua, according to the *Chinese Pharmacopoeia* (2020 version, Chinese Pharmacopoeia Commission, Beijing, China) [1]. The National Health Commission of China approved PCR as a tonic remedy and functional food. PCR is commonly used as a herbal supplement in Asia, Europe, and North America to treat fatigue, weakness, diabetes [2,3], hyperlipidemia [4], and osteoporosis [5]. Consequently, both the consumption and demand for PCR have expanded in recent years. The use of this plant was first recorded in “*Mingyi Bielu*” by Tao Hongjing. According to the theory of traditional Chinese medicine (TCM), its efficacy is derived by supplementing qi, nourishing yin, replenishing the kidney system, and improving the strength of the spleen and lungs. Raw PCR exhibits a certain degree of irritation, so it is necessary to process raw PCR based on the TCM theory. The main purpose of processing is to eliminate irritation and enhance the effect. According to the *Chinese Pharmacopoeia* (2020 version, Chinese Pharmacopoeia Commission, Beijing, China), raw PCR is processed by steaming with rice wine [1].

The main components in PCR include cholestanol saponins, furostanol saponins, spirostanol saponins, triterpenoid saponins, flavonoids, oligosaccharides, polysaccharides, and amino acids [6]. Previous reports showed that PCR saponins exhibit antitumor [7], antidiabetic [3], anti-inflammation [6], antiosteoporotic [8,9], and neuroprotective pharmacological activities [6]. Homoisoflavonoids in PCR exhibit antidiabetic and antitumor pharmacological activities [10]. Oligosaccharides are also important functional ingredients in PCR. Previous research revealed that oligosaccharides hydrolyzed from polysaccharides in PCR exhibit remarkable antiherpetic bioactivity [11], and oligosaccharides isolated from PCR possess significant anti-inflammatory activity [12]. Polysaccharides are believed to be one of the major active ingredients in PCR [13]. Quality control of PCR is based on the polysaccharide content, and the quality control was defined as the lowest content of 7% in raw PCR according to the *Chinese Pharmacopoeia* (2020 version, Chinese Pharmacopoeia Commission, Beijing, China) [1]. Therefore, numerous studies have focused on polysaccharides in recent years, and the polysaccharides in PCR exhibit immunity-enhancement [14], antiosteoporosis [15], and antidiabetic effects [16]. These components collectively contribute to PCR activities. Furthermore, mucilage, calcium oxalate crystal, and volatile components (hexanal and camphene) may be responsible for the irritation caused by of raw PCR [17], but current studies lack evidence to support this speculation. The levels of some chemical constituents increase or decrease during processing, and these changes might be responsible for the enhanced efficacy of processed PCR [18,19,20,21]. Therefore, it is important to distinguish the processing state of PCR for safer and more effective use. In recent years, PCR has been widely available in herbal and nutraceutical markets. However, no comprehensive studies have been conducted on secondary metabolites, monosaccharides, oligosaccharides, or polysaccharides during processing, and there is a lack of reliable chemical data to support the relationship between processing stage and chemical variations during PCR processing. Thus, secondary metabolites and carbohydrates should be taken into account for holistic chemical composition characterization, and it is imperative to study processing-induced chemical variations in PCR to control the PCR processing stage.

Metabolomics and glycomics are two important branches of chemomics. Metabolomics seeks to dynamically and holistically elucidate small biomolecule variations [22,23,24]. Glycomics aims to perform qualitative and quantitative analysis of carbohydrates, including monosaccharides, oligosaccharides, and polysaccharides [25,26]. Currently, the strategy of integrating metabolomics and glycomics is widely used to elucidate overall chemical characteristics of functional food and herbal medicines [27,28,29]. PCR contains a wide variety of secondary metabolites, monosaccharides, oligosaccharides, and polysaccharides [18]. With respect to the detection of small biomolecules, ultraperformance liquid chromatography tandem quadrupole time-of-flight mass spectrometry (UPLC-Q-TOF-MS/MS) has the advantage of high sensitivity, and the analytical technique is well-developed to screen differential metabolites during processing [30,31,32]. Various analytical technologies are needed to obtain all sorts of parameters of polysaccharides because polysaccharides are macromolecular, with complex multidimensional structures. Some chemical structure modifications of polysaccharides, such as hydrolysis or derivatization, are imperative before chromatographic or MS analysis. Ultraviolet detection (UV), refractive index detection (RID), and evaporative light-scattering detection (ELSD) are common analytical techniques for the structural elucidation of polysaccharides [27,28,29]. Integration of metabolomics and glycomics represents a promising approach to reveal the relationship between processing stage and chemical variations.

In the present study, the overall chemical variations in PCR during processing were elucidated by integrating metabolomics and glycomics. The study was designed as follows. First, PCR materials steamed for 0, 1, 2, 3, 4, 6, 8, 10, 12, 14, 16, and 18 h were prepared. Second, the secondary metabolites, monosaccharides, and oligosaccharides of various PCR materials were determined by UPLC-QTOF-MS/MS, whereas the qualitative and quantitative PCP information was investigated by multiple chromatographic techniques. A precolumn derivatization method for high performance liquid chromatography-photo diode array (HPLC-PDA) was applied to characterize variations in monosaccharide composition. A high-performance gel permeation chromatography-refractive index detector (HPGPC-RID) was used to determine the molecular weight of PCP. An ultraviolet (UV) detector was utilized to measure the polysaccharide, uronic acid, and glycoprotein contents of PCP during processing. Third, in order to distinguish PCR in different processing stages, multivariate statistical analysis was conducted to screen differential markers from secondary metabolites, monosaccharides, and oligosaccharides during processing, and multiple chemical parameters of polysaccharides were obtained and analyzed. Thus, the component variations in PCR were explained from a holistic perspective. This research provides a scientific basis to support the relationship between processing stage and chemical variations in PCR. The distinction of processing stage will contribute to safer and more effective use of PCR.

## 2. Results and Discussion

### 2.1. Method Optimization and Validation

Before MS data acquisition for metabolomics and glycomics study, 100 μL of each methanol extract of secondary metabolomics was combined to provide a quality control (QC) sample for metabolomics. Similarly, 100 μL of each 80% methanol extract of monosaccharides and oligosaccharides was combined to provide a quality control (QC) sample for glycomics. The QC samples were run prior of the main analytical sequence six times per day, and the QC samples were injected between ten PCR samples for further quality assurance. The precision, stability, and repeatability of the methods were verified, and the RSD values were less than 3.0% (Supplementary Materials (Section S1, Tables S1–S4)). All the results showed that the established methods were linear, sensitive, precise, accurate, and stable. These methods could be used for the qualitative and quantitative analysis of the secondary metabolites, oligosaccharides, monosaccharides, and polysaccharides in PCR samples.

### 2.2. Compound Identification of Secondary Metabolites, Oligosaccharides, and Monosaccharides

**Figure 1:**
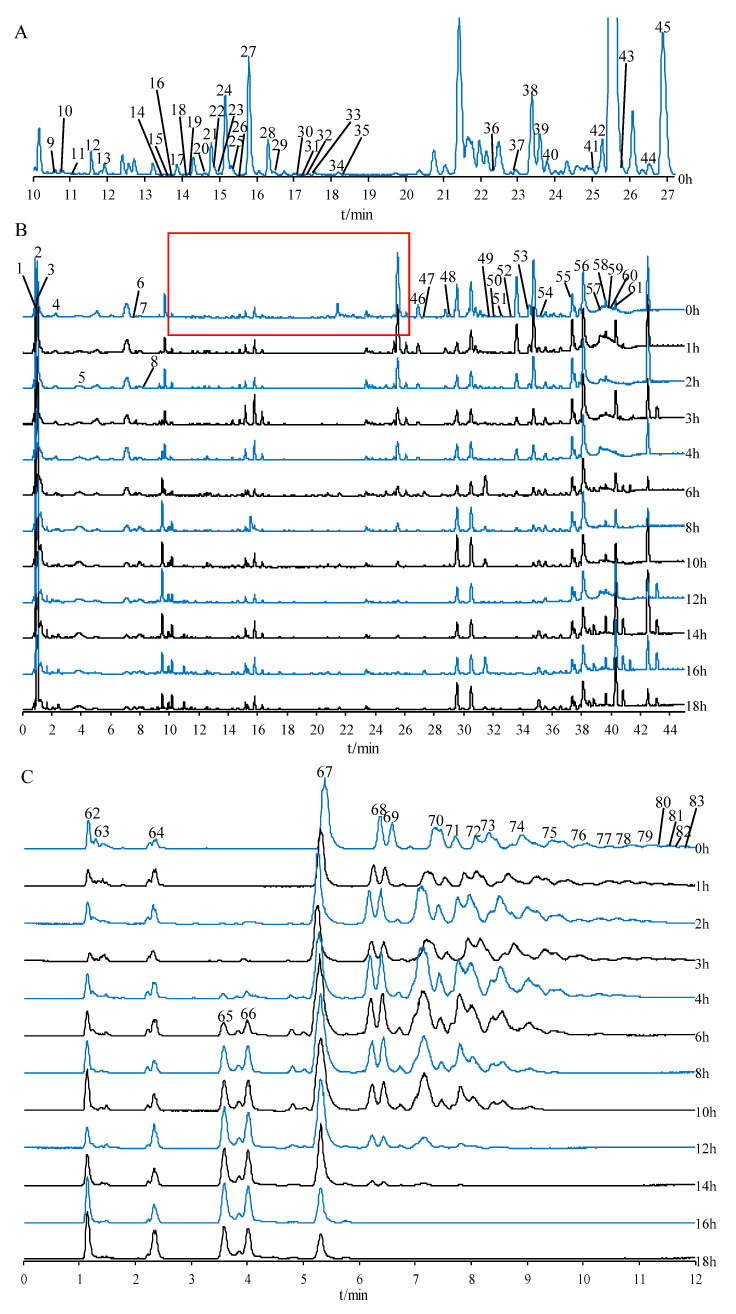
(**A**) Base peak intensity (BPI) chromatogram of secondary metabolites in raw *Polygonatum cyrtonema* rhizome from 10 to 27 min. (**B**) BPI chromatograms of secondary metabolites in PCR with varying processing times. (**C**) BPI chromatograms of oligosaccharides and monosaccharide derivatives in PCR with varying processing times.

In this research, the chemical components in PCR with varying processing times were investigated by UPLC-QTOF-MS/MS, and peak identification was performed. A total of 83 compounds, including 3 cholestanol saponins, 4 furostanol saponins, 30 spirostanol saponins, 1 triterpenoid saponin, 17 flavonoids, 21 oligosaccharides or monosaccharide derivatives, 1 amino acid, and 6 other compounds, were identified or tentatively assigned by UNIFY 1.7 software (Waters Corporation, Milford, CT, USA) by matching of empirical molecular formulae, quasi-molecular ions, and fragment ions. The base peak intensity (BPI) chromatogram of secondary metabolites in raw PCR from 10 to 27 min is shown in Figure 1A, and the chromatograms of secondary metabolites with varying processing times in positive mode are shown in Figure 1B. The chromatograms of oligosaccharides and monosaccharides in negative mode are shown in Figure 1C, and the identified compounds are listed in Table 1.

#### 2.2.1. Identification of Saponins

**Figure 2:**
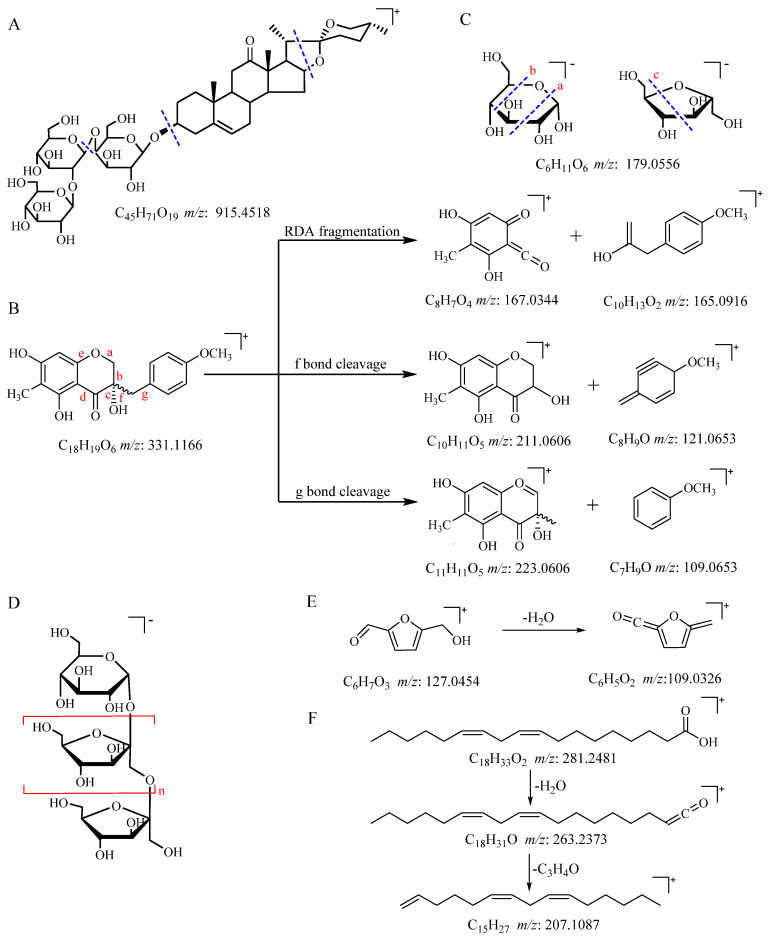
(**A**) Fragmentation regularity of pratioside D_1_. (**B**) (3S)-3,5,7-trihydroxy-6-methyl-3-(4′-methoxybenzyl) chroman-4-one Glucose and (**C**) D-fructose. (**D**) Inulin-type oligosaccharides (**E**) 5-Hydroxymethylfurfural and (**F**) Linoleic acid.

A total of 38 saponins were identified in raw PCR and processed PCR; saponins were the most numerous components in identified small molecules. When saponins were bombarded with high energy, characteristic fragment ions were produced by the loss of a series of glycosyl and internal cleavage of sapogenin [33]. For example, two steroidal saponins, compounds 20 and 23, exhibited [M+H]^+^ ions at *m*/*z* 915.4518 and *m*/*z* 753.4026 in positive mode, respectively. The fragment ions of [M+H-nC_6_H_10_O_5_]^+^ were detected at *m*/*z* 591.3515 and *m*/*z* 429.2989. The fragment ion at *m*/*z* 297.2206 was obtained by sapogenin internal cleavage. Compounds 20 and 23 were tentatively characterized as pratioside D_1_ and (25S)-kingianoside A. The specific cleavage bond of pratioside D_1_ is shown in Figure 2A.

#### 2.2.2. Identification of Flavonoids

A total of 17 flavonoids, including 15 homoisoflavonoids and 2 flavonols, were identified in positive mode. Retro–Diels–Alder (RDA) reaction and hydrogen rearrangement in the C ring of flavonoids occurred as a result of high-energy collisions. Methylene bond cleavage between ring B and ring C formed characteristic fragment ions of homoisoflavonoids. For example, compound 55 had the molecular ion [M+H]^+^ at *m*/*z* 331.1166. The fragment ions at *m*/*z* 167.0344 and *m*/*z* 165.0916 could be attributed to the RDA cleavage of a, c bonds. The characteristic fragment ions at *m*/*z* 211.0606 and *m*/*z* 121.0653 were obtained by α cleavage at the f bond, and the fragment ions at *m*/*z* 223.0606 and *m*/*z* 109.0653 were generated by α cleavage at the g bond. The fragmentation regularity of (3S)-3,5,7-trihydroxy-6-methyl-3-(4′-methoxybenzyl) chroman-4-one is shown in Figure 2B.

#### 2.2.3. Identification of Oligosaccharides and Monosaccharides

Figure 1C shows that with the extended processing time, oligosaccharides with a high degree of polymerization gradually hydrolyzed. Fructo-oligosaccharides (FOS) in PCR are mainly inulin-type oligosaccharides [11,34]. Inulin-type oligosaccharides are linear chains formed by (1→2)-β-D-fructofuranoside bonds. One end of the carbohydrate chain is sucrose. The radical of β-furan-type fructose repeatedly appeared in the middle of the chain, and the end of the chain is a glucose residue [11,34]. Monosaccharides and oligosaccharides were detected in negative mode. Compounds 62, 67, 68, 69, and 70 were identified as D-fructose, sucrose, 1-kestose, raffinose, and nistose, respectively, by comparing the retention time and fragment ions of reference standards.

The cleavage patterns of monosaccharides, such as glucose and D-fructose, were as follows: the two monosaccharides had the molecular ion at *m*/*z* 179 [M-H]^−^, and secondary the mass-spectrogram had fragment ion peaks at *m*/*z* 161 [M-H-18]^−^, 143 [M-H-36]^−^, 119 [M-H-60]^−^, and 89 [M-H-90]^−^. Secondary mass spectrometry analysis was used to compare the mass differences between the molecular ions and fragment ions. *m*/*z* 161 was formed by dehydration of the molecular ion. The bond cleavage of the molecular ion in position ‘a’ produced a fragment ion peak at *m*/*z* 119, and the bond cleavage in position ‘b’ generated a fragment ion peak at *m*/*z* 89. The fragmentation patterns are shown in Figure 2C. Compounds 65 and 66 had the same fragment ions at *m*/*z* 179, 161, 143, 113, and 101, implying the presence of hexose. The fragment ion at *m*/*z* 323 resulted from [2M-H-H_2_O]^−^, so compound 65 and 66 could be fructose or glucosyl residues.

Oligosaccharides also follow the MS fragmentation regularity of monosaccharides, such as glucose or fructose. Molecular ion peaks, such as [M-H]^−^ and [M+HCOO]^−^, were detected in low-energy MS. High-energy MS produced fragments of [M-H-18]^−^, [M-H-36]^−^, [M-H-60]^−^, and [M-H-90]^−^. In addition, the cleavage of the O-glycoside bond of oligosaccharides induced the characteristic fragmentation ions of [M-H-n162]^−^ and [M-H-n180]^−^. The FOS polymerization degrees in raw PCR detected in this study ranged from 2 to 15. With increase polymerization degrees, the increased radical and connection mode remained unchanged. The structure is shown in Figure 2D.

#### 2.2.4. Identification of Other Compounds

TCM with high saccharide content produces 5-HMF after thermal processing. The molecular ion of compound 5 at *m*/*z* 127.0454 [M+H]^+^ lost a molecule of H_2_O. Then, the fragment ion at *m*/*z* 109.0326 was obtained, which was consistent with the fragment ion of the reference substance. Figure 2E shows the fragmentation regularity of 5-HMF. Organic acids are long carbon chains with carboxyl groups, and characteristic fragment ions are easily cracked at carboxyl groups. Organic acids have multiple unsaturated bonds, so they are susceptible to Mclafferty rearrangement. For example, compound 56 was identified as linoleic acid, and [M+H]^+^ lost a molecule of H_2_O at the carboxylate group to generate a fragment ion at *m*/*z* 263.2373 [M+H-H_2_O]^+^ in positive ion mode. Then, Mclafferty rearrangement occurred to provide the fragment ion at *m*/*z* 207.1087 [M+H-C_3_H_4_O] ^+^. The specific fragmentation pattern is shown in Figure 2F.

### 2.3. Screening of Differential Markers from Secondary Metabolites, Oligosaccharides, and Monosaccharides

**Figure 3:**
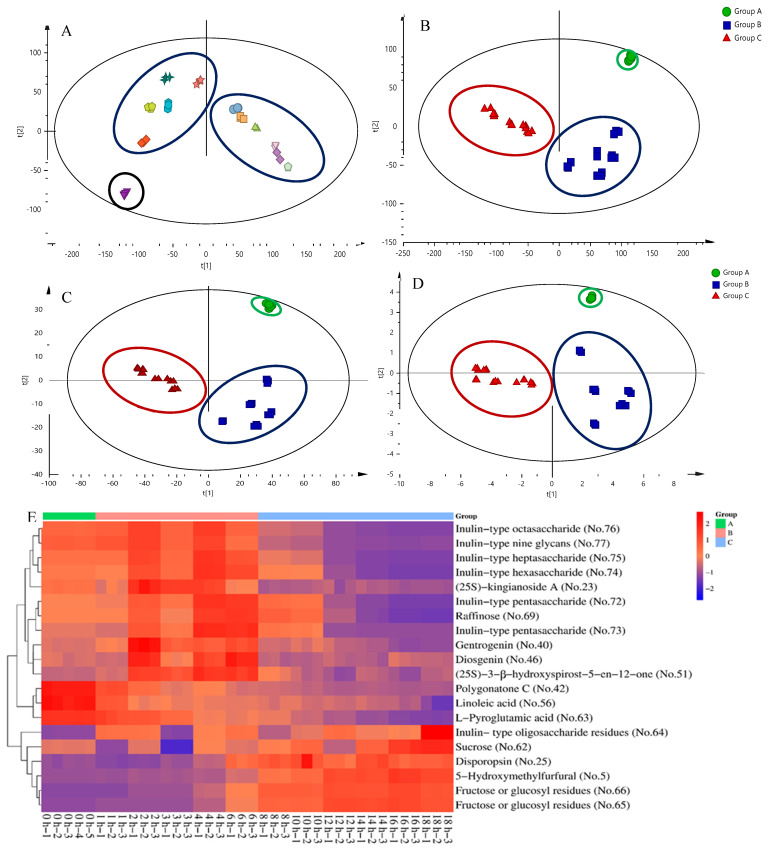
(**A**) PCA plot of 38 samples depending on 14,772 variables; R^2^X = 0.92, Q^2^ = 0.834. (**B**) PLS-DA plot of 14,772 variables; R^2^X = 0.79, R^2^Y = 0.996, Q^2^ = 0.989. (**C**) PLS-DA plot of 1846 variables; R^2^X = 0.864, R^2^Y = 0.991, Q^2^ = 0.980. (**D**) PLS-DA plot of 20 differential markers; R^2^X = 0.886, R^2^Y = 0.920, Q^2^ = 0.911. (**E**) Heat map of 20 differential markers. Raw PCR, PCR processed for 1–6 h and PCR processed for 8–18 h were clustered into groups A, B, C, respectively.

MS data of secondary metabolomics, monosaccharides, and oligosaccharides were preprocessed by Progenesis QI 2.3 software (Waters Corporation, Milford, CT, USA), and 14,772 variables were detected and listed in an Excel table. The variable information was imported into Simca-P 14.1 software. The PCA results were demonstrated by score plots to visualize the extent of aggregation and separation among different sample groups through dimension reduction in complicated data; therefore, PCA was first applied to check the natural grouping properties and suspicious values of original variables [35,36,37]. As shown in Figure 3A, all samples fell well within the 95% confidence interval. The goodness of fit (R^2^X = 0.92) was close to 1. The goodness of prediction (Q^2^ = 0.834) exceeded 0.5, and the difference was within 0.2, indicating satisfactory quality of the PCA model. Raw PCR, PCR processed for 1–6 h, and PCR processed for 8–18 h were clustered into Groups A, B, and C, respectively, indicating that the processing of PCR could be divided into three stages, namely the raw stage (0 h), the middle stage (1–6 h), and the late stage (8–18 h). Because PLS-DA is more effective than PCA in searching for differential metabolites, supervised PLS-DA was used to identify differential markers among the processing groups of PCR [38]. The variable importance parameter (VIP) value denotes the contribution of each variable to the PLS-DA model [29]. A total of 38 batches of samples were grouped by PLS-DA, and 1846 variables with VIP values > 1 and one-way ANOVA test *p* < 0.05 were selected. A total of 20 compounds were identified among the 1846 variables. PLS-DA models based on 14,772 variables, 1846 variables, and 20 differential markers were established. The results are shown in Figure 3B–D, respectively. The PLS-DA models based on different variables were all satisfactory, with significant distinction effects between the three groups. The score plots showed good separation between the three groups (14772 variables, R^2^X = 0.79, R^2^Y = 0.996, Q^2^ = 0.989, Figure 3B; 1846 variables, R^2^X = 0.864, R^2^Y = 0.991, Q^2^ = 0.980, Figure 3C; 20 markers, R^2^X = 0.886, R^2^Y = 0.920, Q^2^ = 0.911, Figure 3D). Therefore, the 20 screened differential markers, including one furfural derivative (compound 5), four spirostanol saponins (compounds 23, 40, 46, and 51), two flavonoids (compounds 25 and 42), one fatty acid (compound 56), one monosaccharide (compound 62), one amino acid (compound 63), eight oligosaccharides (compounds 64, 70, 72, 73, 74, 75, 76, and 77), and two monosaccharide residues (compounds 65 and 66) could replace the original 14,772 variables to distinguish PCR of different processing stages. These differential markers might contribute to the therapeutic variations in PCR of different processing stages. In the future, pharmacology could be integrated to further reveal the relationship between differential markers and therapeutic changes. A heat map of 20 differential markers of secondary metabolites, oligosaccharides, and monosaccharides is shown in Figure 3E.

### 2.4. UPLC-QTOF-MS/MS Analysis of Secondary Metabolites, Oligosaccharides, and Monosaccharides

**Figure 4:**
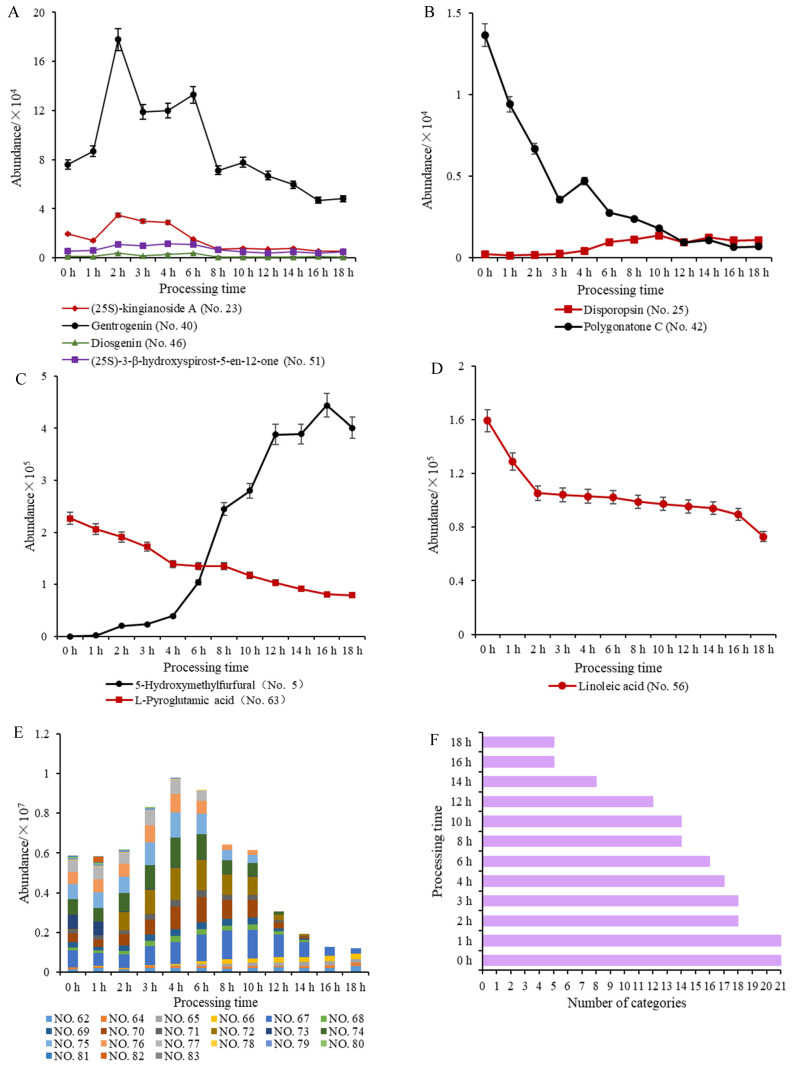
(**A**) Average intensity of saponin markers. (**B**) Flavonoid markers. (**C**) 5-HMF and L-pyroglutamic acid. (**D**) Linoleic acid. (**E**) Oligosaccharides and monosaccharide derivatives. (**F**) Number of oligosaccharides and monosaccharide derivatives during processing.

Spirostanol saponins and flavonoids accounted for the majority of the identified secondary metabolites. Saponins are sensitive in high-temperature environments, and the glycosidic bond can be hydrolyzed to release the monoglycosyl or oligosaccharyl. The differential markers of spirostanol saponins, including (25S)-kingianoside A, gentrogenin, diosgenin, and (25S)-3-β-hydroxyspirost-5-en-12-one, showed a tendency to first increase and then decrease (*p* < 0.05) during processing, indicating that multiple chemical reactions occurred in succession (Figure 4A). First, the hydrolysis of glycosidic bonds caused an increase in sapogenins during the middle processing stage (1–6 h). Second, mutual transformation resulted in a decrease during the late processing stage (8–18 h). With respect to differential markers of flavonoids, disporopsin first increased and then decreased (*p* < 0.05), and polygonatone C decreased (*p* < 0.05) (Figure 4B). The hydrolysis of flavonoid glycosides and the transformation of flavonoid aglycones could be responsible for the results. 5-HMF is a saccharide derivative produced by dehydration of hexose (usually glucose and fructose) under acidic and thermal conditions. 5-HMF is a marker of a Maillard reaction in the processing of various herbal medicines and foods [39]. 5-HMF gradually increased (*p* < 0.05), and L-pyroglutamic acid decreased (*p* < 0.05). The results suggest that a Maillard reaction occurred between sugars and amino acids to produce furfural derivatives (Figure 4C). In addition, with extended processing time, the intensity of linoleic acid was reduced (*p* < 0.05), indicating that an oxidative decomposition reaction occurred under high-temperature conditions (Figure 4D). These chemical differences might contribute to the therapeutic variations in PCR with varying processing times. PCR processing entered a new stage after 6 h, and the chemical characterization of PCR changed significantly from the middle to late processing stage.

Compared with raw PCR, processed PCR became clearly dark-colored, especially in the late processing stage (8–18 h). This phenomenon is closely related to carbohydrate variations during PCR processing. UPLC-Q-TOF-MS/MS analysis showed that 21 oligosaccharides and monosaccharide derivatives were detected in raw PCR (Figure 4E,F). Monosaccharides and their derivatives containing D-fructose and three saccharide residues showed an increasing tendency (*p* < 0.05) during processing (Figure 4E). Fructose significantly increased (*p* < 0.05) after processing, indicating that fructose is an important component of PCR polysaccharides and oligosaccharides. The taste of processed PCR was sweeter than that of raw PCR, which was associated with the significant increase in fructose [40]. Oligosaccharides increased significantly (*p* < 0.05) in the middle processing stage and decreased sharply (*p* < 0.05) in the late processing stage (Figure 4E). The total response intensities were highest after 4 h (Figure 4E). The results illustrate that the polysaccharides were mainly hydrolyzed into oligosaccharides in the middle processing stage. With extended processing time, the PCR processing entered the late stage, and the PCR oligosaccharides with a high degree of polymerization (DP 3–15) were gradually hydrolyzed into monosaccharides, disaccharides, and their derivatives at high temperature. The types of oligosaccharides were continuously reduced (*p* < 0.05), and only five types of saccharides, including D-fructose, sucrose, and three saccharide residues, were detected in PCR processed for 16 and 18 h (Figure 4E,F). Furthermore, the decrease in total oligosaccharides illustrated that processed PCR mainly underwent hydrolysis and Maillard reaction during the late processing stage. The melanoidins produced by the Maillard reaction between sugars and proteins under thermal conditions was primarily responsibility for the dark-colored appearance of processed PCR [41].

### 2.5. Qualitative and Quantitative Analysis of PCP during Processing

#### 2.5.1. Monosaccharide Composition Variations in PCP during Processing

**Figure 5:**
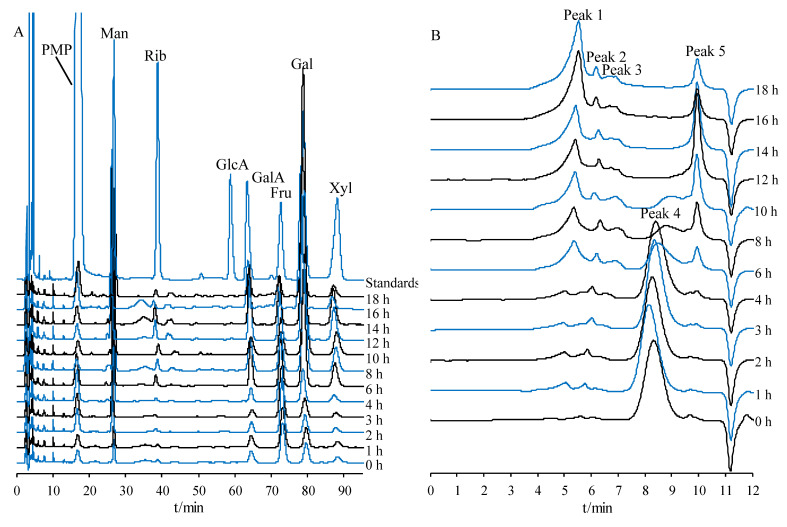

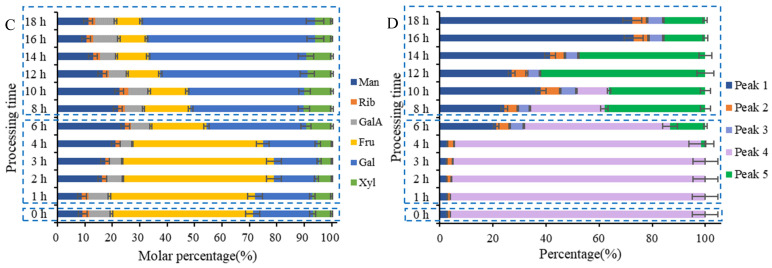

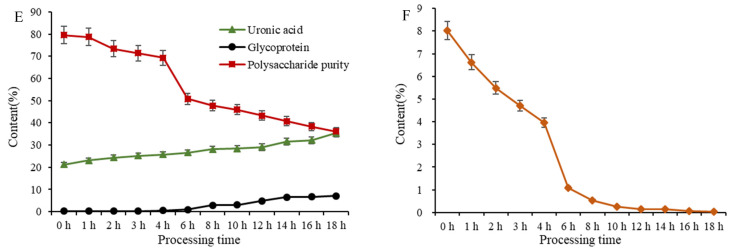
(**A**) Monosaccharide composition of PCP with varying processing times. (**B**) HPGPC-RID chromatograms of PCP with varying processing times. (**C**) The molar ratio of each monosaccharide to all monosaccharides. (**D**) Ratio of each PCP peak area to total PCP peak area. (**E**) Glycoprotein, uronic acid, and total polysaccharide content variations of PCP during processing. (**F**) Polysaccharide content variations of PCR medicinal materials during processing.

The results of monosaccharide composition analysis showed that the polysaccharides of different PCR samples were mainly composed of six kinds of monosaccharides, including Man, Rib, GalA, Fru, Gal, and Xyl (Figure 5A), although the mole ratios differed among samples (Figure 5C). Fru and Gal accounted for the largest proportion of PCP. Fru accounted for the highest proportion of raw PCP, Fru > Gal (*p* < 0.05). The proportion of Fru gradually decreased (*p* < 0.05), and Gal significantly increased (*p* < 0.05) during processing. The proportion of Man and Xyl both showed an increasing trend before decreasing (*p* < 0.05). The variation patterns of Rib and GalA were not obvious during processing, which could be explained by two aspects. First, polysaccharides hydrolyzed into oligosaccharides and/or monosaccharides. For example, the decrease in the Fru ratio in PCP was related to the hydrolysis of polysaccharides to inulin-type oligosaccharides and Fru. Second, the acid hydrolysis efficiency of PCP could be further affected by processing-induced chemical structure variations, therefore, the proportion of released monosaccharides would change [42,43]. The monosaccharide composition of PCP changed significantly after processing for 6 h. Gal accounted for the highest proportion, Gal > Fru (*p* < 0.05), indicating that the chemical properties of PCP were altered remarkably during different processing stages. 

#### 2.5.2. Molecular Weight Variations of PCP during Processing

Polysaccharides are abundant in PCR and exhibit multiple biological activities. The molecular weight of PCP changed significantly (*p* < 0.05) during processing. HPGPC chromatograms of PCP with varying processing times are shown in Figure 5B. Based on the established lgM_w_-RT calibration curve (lgM_w_ = −0.7229 t + 9.3918, R^2^ = 0.9959), one distinct peak was observed at 8.396 min (2100.54 Da, peak 4) in Raw PCP. The polysaccharides of peak 4 gradually converted to polysaccharides of peak 1 (5.234 min, 405640.58 Da), peak 2 (5.938 min, 125666.58 Da), and peak 3 (6.827 min, 28612.89 Da) after processing. We speculated that a Maillard reaction occurred between polysaccharides of 2100.54 Da and proteins; then, new polysaccharides of higher molecular weight appeared. Furthermore, peak 5 with a low molecular weight (9.935 min, 162.10 Da) was generated after steaming for 6 h because the polysaccharides were partially hydrolyzed into monosaccharides and Maillard reaction derivatives by heating. Maillard reaction and hydrolysis of polysaccharides produced new molecular-weight saccharides that might be responsible for the variations in polysaccharide bioactivities after processing [42]. The peak area percentages of polysaccharides of different molecular weights are shown in Figure 5D. Peak area percentages changed significantly (*p* < 0.05) after processing for 6 h, suggesting that chemical properties of PCP were remarkably altered. The aforementioned molecular weight and monosaccharide composition variations revealed that the chemical transformation stages of PCP were consistent with secondary metabolites, oligosaccharides, and monosaccharides. The PCP chemical transformation could also be roughly divided into three stages, namely the raw stage (0 h), the middle stage (1–6 h), and the late stage (8–18 h). The chemical variations of all kinds of components entered a new stage after processing for 6 h, indicating the inherent relevance of carbohydrates and secondary metabolites.

#### 2.5.3. Glycoprotein, Uronic Acid, and Total Polysaccharide Content Variations of PCP during Processing

Calibration curves and sensitivity of the glycoprotein, uronic acid and total polysaccharide determination assays were shown in Table S5. The basic physicochemical properties of with varying processing times of PCP showed significant differences (Figure 5E). The total polysaccharide content of crude PCP showed a decreasing trend during processing (*p* < 0.05), indicating that the polysaccharide purity gradually decreased. The total polysaccharide content of PCR medicinal materials decreased during processing (*p* < 0.05), indicating that the polysaccharides were mainly hydrolyzed into oligosaccharides, monosaccharides, and their derivatives after processing for 18 h (Figure 5F). The uronic acid content showed an increasing trend, suggesting that the content of acid polysaccharides increased (*p* < 0.05) after processing. The increase was related to the Maillard reaction between amino acids and saccharides. The increased (*p* < 0.05) glycoprotein content might be related to the increase in the number of chemical bonds between proteins and polysaccharides in the late processing stage. The results verified that a Maillard reaction occurred under heating conditions.

### 2.6. Processing-Induced Chemical Mechanisms

**Figure 6:**
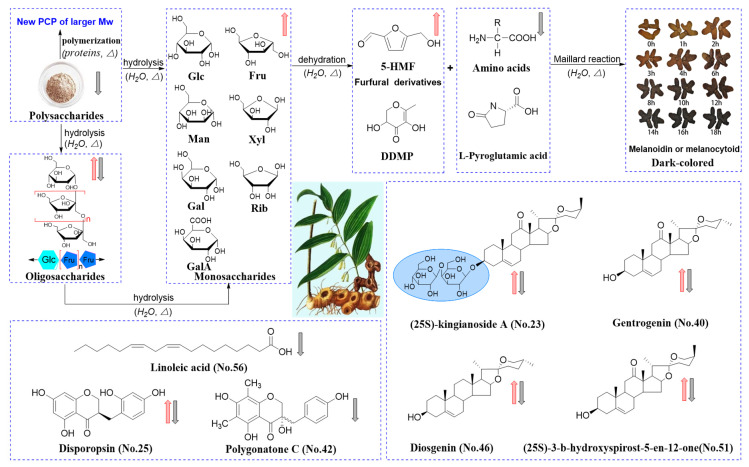
Processing-induced chemical variations in PCR.

In general, the processing-induced chemical reactions in PCR were mainly influenced by temperature and water. Multiple chemical mechanisms, including hydrolysis, oxidative decomposition, dehydration, Maillard reaction, and polymerization, were involved in the processing and caused qualitative and quantitative changes in chemical ingredients (Figure 6). Concretely, fatty acids might involve an oxidative decomposition reaction. In the middle processing stage, the glycosidic bonds in saponins, flavonoid glycosides, oligosaccharides, and polysaccharides could be hydrolyzed under acidic and thermal conditions. The hydrolysis and dehydration reactions could release monosaccharides and their derivatives [27,28]. In the late processing stage, further hydrolysis of polysaccharides, oligosaccharides, and monosaccharides could produce DDMP and furfural derivatives, such as 5-HMF; then, the produced ingredients and amino acids would undergo a Maillard reaction under thermal conditions, indicating the inherent relevance of carbohydrates and secondary metabolites [18,44]. The products of Maillard reactions, such as melanoidin or melanocytoid, might cause variation in PCR [18,44]. Furthermore, some polysaccharides polymerize with proteins to produce new polysaccharides of higher molecular weight during processing. These chemical transformations might contribute to the therapeutic variations in PCR with varying processing times.

## 3. Materials and Methods

### 3.1. Reagents, Chemicals, and Materials

Trifluoroacetic acid (TFA), formic acid (MS grade), and potassium phosphate monobasic (HPLC grade) were supplied by Macklin (Shanghai, China). 1-Phenyl-3-methyl-5-pyrazolone (PMP) was obtained from Yuanye Biotechnology Co., Ltd. (Shanghai, China). Methanol (HPLC grade), acetonitrile (HPLC and MS grade), and ammonium hydroxide solution (MS grade) were obtained from Thermo Fisher Scientific (Shanghai, China). Chloroform, sodium hydroxide, hydrochloric acid, sulfuric acid, acetic acid, and anthrone were purchased from Sinopharm Chemical Reagent Co., Ltd. (Beijing, China). Coomassie brilliant blue G250, albumin bovine Ⅴ, and carbazole were purchased from Biotopped Co., Ltd. (Beijing, China). Rice wine was supplied by Jinfeng Wine Co., Ltd. (Shanghai, China). The alcohol content (20 °C) of the rice wine used for steaming was 14% vol.

Reference substances, including D-fructose (Fru), D-glucose (Glc), D-galactose (Gal), D-mannose (Man), D-galacturonic acid (GalA), D-glucuronic acid (GlcA), D-xylose (Xyl), D-Ribose (Rib), sucrose, 1-kestose, raffinose, and nistose, were purchased from Yuanye Biotechnology Co., Ltd. (Shanghai, China). 5-Hydroxymethylfurfural (5-HMF), rutin, (20α,22R,25S)-spirosta-5-ene-3β-ol, and kaempferol were obtained from Chroma-Biotechnology Co., Ltd. (Chengdu, China). 2, 3-dihydro-3, 5-dihydroxy-6-methyl-4(H)-pyran-4-one (DDMP) was produced by TLC Pharmaceutical Standards Ltd. (Toronto, ON, Canada). Dextran reference substances with known molecular sizes (180 Da–300.6 KDa) were supplied by the National Institute for the Control of Pharmaceutical and Biological Products (Beijing, China). The purity of all chemical standards was above 98%.

Raw PCR samples collected from Yongzhou, Hunan, were authenticated by Professor Zhuju Wang. The quality of the herbal samples was checked against the *Chinese Pharmacopoeia* (2020 version, Chinese Pharmacopoeia Commission, Beijing, China) [1]. All herbal samples were qualified. Voucher specimens were deposited at the Institute of Chinese Materia Medica, China Academy of Chinese Medical Sciences, Beijing. Plant material collection information is summarized in Supplementary Materials (Section S2, Table S6).

### 3.2. Sample Preparation

#### 3.2.1. Sample Preparation with Varying PCR Processing Times

The processing time lasted 0–18 h according to three factors. “*Lei Gong Pao Zhi Lun*”, written by Lei Xiao, is the earliest monograph on Chinese medicine processing. The processing methods and technologies recorded in “*Lei Gong Pao Zhi Lun*” provide ideas and references with respect to PCR processing. The processing time recorded in the book is from morning (period of the day from 9 a.m. to 11 a.m.) to midnight (period of the day from 11 p.m. to 1 a.m.), and the steaming time was listed as nearly 14 h. After steaming for 18 h, the polysaccharide content in the PCR medicinal materials decreased obviously. Polysaccharides were mainly hydrolyzed. Color change is correlated with processing degree and internal quality. Traditional experience suggests that a black color is preferable when PCR is repeatedly steamed and dried during processing. Therefore, the processing times used in this research could refer to sample color. The sample color no longer changed after 18 h of processing, so the processing time was set to 0–18 h. Because the internal and external quality of PCR changed obviously during 0–4 h of processing, the sampling points were more intensive during this period. The *Chinese Pharmacopoeia* (2020 version, Chinese Pharmacopoeia Commission, Beijing, China) describes the processing method for raw PCR as follows: ten kg raw PCR infiltration with 2 kg rice wine for 12 h; then, steaming for 0, 1, 2, 3, 4, 6, 8, 10, 12, 14, 16, and 18 h, followed cutting into thick slices and drying at room temperature [1]. Raw PCR samples were prepared in quintuplicate batches, and processed PCR samples were prepared in triplicate. All PCR samples were crushed into powder and sieved through a sieve of 80 mesh (0.18 mm).

#### 3.2.2. Secondary Metabolomics Sample Preparation

A total of 38 PCR samples were analyzed (5 × raw samples; 3 × samples each processed for 1, 2, 3, 4, 6, 8, 10, 12, 14, 16, and 18 h). Dried PCR powder (1 g) was extracted by reflux with 20 mL methanol for 30 min. Ten mL extract solution of each sample was blow-dried using nitrogen gas. The residue was redissolved using 1 mL methanol and filtered with a 0.22 µm syringe filter for UPLC-QTOF-MS/MS analysis of secondary metabolomics. All samples were prepared in parallel for the study.

#### 3.2.3. Glycomics Sample Preparation 

PCR powder (0.1 g) was extracted with 25 mL 80% methanol for 40 min, with an ultrasonic extraction power of 250 W. Supernatant was centrifuged at 12,000 rpm for 20 min to perform UPLC-QTOF-MS/MS analysis of monosaccharides and oligosaccharides. The preparation methods of polysaccharides were as follows: PCR powder was extracted to degrease and remove small molecules (1 h × 2 times) with 90% ethanol (1:10, *w*/*v*), and the dried residue powder (50 g) was extracted by reflux using 10 volumes of water (1 h × 2 times). The filtrate was concentrated to 1/4 of the initial volume at 65 °C using a rotary vacuum evaporator (Rotavapor R-3 BUCHI). Then, 95% ethanol was slowly added to the concentrated liquor until the concentration reached to 80% (*v*/*v*) and kept at 4 °C for 12 h.

The ethanol precipitation was collected and dissolved with water. Sevage reagent was used to remove free protein; then, polysaccharide solution was dialyzed by a dialysis bag (3 × 10^3^ Da) and freeze-dried to obtain crude PCP. Exactly 20 mg PCP powder was weighed and dissolved in 2 mL of 20% methanol. After centrifugation at 12,000 rpm for 5 min, the supernatant was obtained for HPGPC-RID analysis.

The preparation methods of precolumn derivatization samples were as follows: exactly 10 mg PCP powder was weighed and dissolved in 2 mL of 2 M TFA solution in a test tube with a stopper. The crude PCP was hydrolyzed for 7 h at 110 °C, and the PCP hydrolysate was evaporated to dryness at 60 °C. The residue was dissolved using 1 mL methanol. After dissolving and blow drying for three times, remnant TFA was removed. Then, the residue was redissolved with 2 mL of water to obtain PCP hydrolysis solution. The hydrolysis solution was subjected to PMP precolumn derivatization. A volume of 200 µL free monosaccharide solution from the polysaccharides was mixed with 200 µL of 0.6 M sodium hydroxide and 200 µL of 0.5 M PMP in a 5 mL screw-cap vial. The derivatization reaction was carried out at 70 °C for 60 min, and the reaction mixture was cooled to room temperature. Then, 400 µL of 0.3 M hydrochloric acid solution was added to neutralize the reaction solution, and 1 mL chloroform was added to remove redundant PMP reagents. After vigorous shaking and centrifugation (12,000 rpm for 5 min), the chloroform layer was discarded. The operation was repeated three times until the chloroform layer became colorless, and the aqueous layer was used as the test solution. Stand solutions, including Man, Rib, GlcA, GalA, Fru, Gal, and Xyl, were also treated according to the same procedure as that for PMP-HPLC-PDA analysis [28].

### 3.3. Secondary Metabolomics UPLC-QTOF-MS/MS Analysis 

#### 3.3.1. UPLC Conditions

Chromatographic analysis was conducted on a Waters 2998 detector furnished with a quaternary pressure pump, a photodiode array detector, and a column oven. The detection wavelength ranged from 200 to 700 nm. Separation was carried out on an ACQUITY UPLC HSS T_3_ column (2.1 × 100 mm, 1.8 µm). UPLC analysis was performed according to the following gradient elution method. The mobile phase was composed of acetonitrile (A) and 0.1% formic acid water (B). The gradient elution conditions were as follows: 0–5 min, 1% A; 5–10 min, 1%–15% A; 10–20 min, 15%–37% A; 20–30 min, 37%–64% A; 30–40 min, 64%–95% A; 40–45 min, 95% A; 45.01–50 min, 1% A. The column temperature was 35 °C. The injection volume was 2 µL, and the flow rate was 0.25 mL/min.

#### 3.3.2. MS Conditions

A time-of-flight mass spectrometer furnished with an orthogonal electrospray ionization (ESI) source was used to conduct metabolomics analysis. The instrument type was a Xevo G2S Q-Tof. Additional secondary metabolites were detected in positive ion mode, so analysis of secondary metabolites was performed in positive ion mode (Figure S1). The positive ion mode capillary voltage was +3 kV, and the cone voltage was 40 V. The ion source temperature was 120 °C, and the desolvation gas flow rate was 600 L/h. The desolvation gas temperature was 450 °C, and the cone gas flow rate was 50 L/h. The parent ion collision energy was 6 eV, and the fragment ion collision energy was 20–50 eV. The mass scanning range was 50–2500 Da, and the scanning time was 0.2 s. Data acquisition and analysis were carried out using Masslynx 4.1 software (Waters Corporation, Milford, CT, USA).

### 3.4. UPLC-QTOF-MS/MS Analysis of Monosaccharides and Oligosaccharides

#### 3.4.1. UPLC Conditions

Separation was conducted using an XBridge BEH amide column (2.1 mm × 100 mm, 2.5 µm). Mobile phase A was 80% acetonitrile with 0.1% ammonium hydroxide, and mobile phase B was 30% acetonitrile with 0.1% ammonium hydroxide. Separation was achieved at 35 °C using linear gradient elution at a flow rate of 0.15 mL/min: 0–10 min, 90%–30% A; 10–20 min, 30% A; 20.01–40 min, 90% A. The run time of the liquid phase was 40.01 min, and the signal acquisition time was 12 min. The injection volume was 1 µL.

#### 3.4.2. MS Conditions

The instrument type was a Xevo G2S Q-Tof. Saccharides are more likely to carry negative charges during ionization, especially acidic saccharides, which could be ionized to RCOO^−^, so glycomics analysis was performed in negative ion mode. The negative ion mode capillary voltage was −2.5 kV, and the cone voltage was 40 V. The ion source temperature was 120 °C, and the desolvation gas flow rate was 600 L/h. The desolvation gas temperature was 450 °C, and the cone gas flow rate was 50 L/h. The parent ion collision energy was 6 eV, and the fragment ion collision energy was 20–50 eV. The mass scanning range was 50–2500 Da, and the scanning time was 0.2 s.

### 3.5. Multiple Chromatographic Techniques for the Analysis of Polysaccharides

#### 3.5.1. HPGPC-RID Analysis of Polysaccharides

The molecular weight (Mw) of PCP was determined by HPGPC on a Waters 1515–2414 HPLC system coupled with a refractive index detector (RID). Separation was achieved by a Shodex OHpak SB-806M HQ column (300 mm × 8 mm, 13 µm). The detector temperature was 40 °C. Furthermore, 20% methanol was used as a mobile phase at a flow rate of 1 mL/min. The column temperature was 40 °C. The injection volume was 10 µL. Dextran reference substances with known molecular sizes (180, 2700, 5250, 9750, 10,350, 36,800, 64,650, 135,350, and 300,600 Da) were analyzed under the same condition to establish the lgMw-RT calibration curve.

#### 3.5.2. PMP-HPLC-PDA Analysis of Polysaccharides

The monosaccharide composition of PCP was determined by a Shimadzu LC-20AT HPLC-PDA system. Separation was performed on a Thermo Scientific C_18_ column (4.6 mm × 250 mm, 5.0 µm) at 35 °C. Mobile phase A was 0.05 M potassium phosphate monobasic-sodium hydroxide buffer containing 0.3% acetic acid, and mobile phase B was acetonitrile. Gradient elution was performed as follows: 0–43 min, 82.5% A; 43–43.5 min, 82.5%–81.5% A; 43.5–100 min, 81.5% A. Separation was achieved at a flow rate of 1 mL/min, and the UV detection wavelength was 250 nm. The injection volume was 20 µL.

#### 3.5.3. Determination of Protein, Uronic Acid, and Total Polysaccharide Content by UV

The total polysaccharide content of crude PCP was determined by anthrone-sulfuric acid method according to the *Chinese Pharmacopoeia* (2020 version, Chinese Pharmacopoeia Commission, Beijing, China) [1]. The protein content was detected by Coomassie brilliant blue method [45], and the uronic acid content was tested using the carbazole-sulfuric acid method [46].

### 3.6. Data Analysis

MS data processing of secondary metabolomics, monosaccharides, and oligosaccharides, including baseline filtering, retention time correction, peak alignment, missing value processing, and data normalization, was performed using Progenesis QI 2.3 metabolomics data processing software (Waters Corporation, Milford, CT, USA). The peak abundances were exported with tR-*m*/*z* pairs and imported into Simca-P 14.1 (Umetrics, Umea, Sweden). PCA was applied to observe the overall distribution among the PCR samples obtained with varying processing times; then, PLS-DA was used to screen identified differential metabolites to distinguish between the sample groups. Progenesis SDF Studio 1.0 (Waters Corporation, Milford, CT, USA) and Waters UNIFY 1.7 (Waters Corporation, Milford, CT, USA) were used to build the compound database of reported substances in PCR. The self-constructed PCR compound database included 220 secondary metabolomics and 31 oligosaccharides. In combination with compound database and standard comparison, 83 compounds were identified by Waters UNIFY 1.7 (Waters Corporation, Milford, CT, USA), and 20 differential metabolites were screened by Progenesis QI 2.3 (Waters Corporation, Milford, CT, USA) software. All results are denoted as mean ± SD. IBM SPSS Statistics 21 (IBM SPSS Corporation, Chicago, IL, USA) was used for statistical comparison (*t*-test), and a *p*-value *<* 0.05 was deemed statistically significant.

## 4. Conclusions

In this research, an integrated chromatographic strategy based on metabolomics and glycomics was applied to analyze the chemical variations of secondary metabolites, oligosaccharides, and monosaccharides in PCR with varying processing times. The results demonstrate that PCR processing can be divided into three stages, namely the raw stage (0 h), the middle stage (1–6 h), and the late stage (8–18 h), and the 20 screened differential markers can distinguish PCR of different processing stages. The chemical transformation stages of polysaccharides were found to be consistent with secondary metabolites, oligosaccharides, and monosaccharides. The chemical variations of all kinds of components entered a new stage after processing 6 h. The processing-induced chemical transformation mechanisms could involve hydrolysis, oxidative decomposition, dehydration, Maillard reaction, and polymerization. An integrated chromatographic strategy could be used to monitor secondary metabolites and carbohydrates simultaneously to explore the overall chemical characterization variations during the processing of herbal medicines and foods. This research provides a scientific basis to support the relationship between processing stage and chemical variations in PCR. The distinction of processing stages could contribute to safer and more effective use of PCR in the future.

## Figures and Tables

**Table 1:** Identification of secondary metabolites, monosaccharides, and oligosaccharides in PCR.

NO.	RT/min	Component	*m*/*z*	Formula	MS/MS Fragmention	Adducts	C.
1	0.87	Polygonatone B	347.1206	C_18_H_18_O_7_	175.1194, 241.0712	[M+H]^+^, [M+K]^+^	e
2	0.89	(22S)-16β-[(α-L-rhamnopyranosyl)oxy]-3β,22-dihydroxycholest-5-en-1β-yl-α-L-rhamnopyranoside	727.4660	C_39_H_66_O_12_	313.2728	[M+H]^+^	a
3	0.90	(22S)-cholest-5-en-1β,3β,16β,22-tetrol-1-O-α-L-rhamnpyranosyl-16-O-β-D-glucopyranoside	743.4617	C_39_H_66_O_13_	184.0738	[M+H]^+^	a
4	2.02	(25R)-Polygodoside E	593.3590	C_33_H_52_O_9_	325.1237	[M+H]^+^	c
5	4.23	5-Hydroxymethylfurfural (5-HMF)	127.0454	C_6_H_6_O_3_	343.0265, 99.0482, 109.0326	[M+H] ^+^ , [3M+H-2H_2_O]^+^	g
6	7.50	(25S)-3β,24-dihydroxy-spirostan-5-en-12-one-3-O-β-D-glucopyranosyl-(1→2)-β-D-glucopyranosyl-(1→4)-β-D-galactopyranoside	948.4807	C_45_H_70_O_20_	561.2966, 387.1856, 337.1713, 175.1193	[M+NH_4_]^+^	c
7	8.51	5,7-dihydroxy-6-methyl-3-(2′,4′-dihydroxybenzyl)-chroman-4-one	317.1113	C_17_H_16_O_6_	310.1280, 141.9589, 195.0657	[M+H]^+^	e
8	8.96	2,3-dihydro-3,5-dihydroxy-6-methyl-4(H)-pyran-4-one(DDMP)	145.0537	C_6_H_8_O_4_	109.0326, 81.0361	[M+H]^+^	g
9	10.77	(E)-3-(3′,4′-dihydroxybenzylidene)-5,7-dihydroxy-8-methoxy-6-methylchroman-4-one	362.1216	C_18_H_16_O_7_	331.1273	[M+NH_4_]^+^	e
10	10.82	(E)-5,7-dihydroxy-6,8-dimethyl-3-(4′-hydroxybenzylidene)-chroman-4-one	330.1319	C_18_H_16_O_5_	205.0969	[M+NH_4_]^+^	e
11	11.04	(3R)-5,7-dihydroxy-6-methoxyl-8-methyl-3-(2′,4′-dihydroxybenzyl)-chroman-4-one	364.1386	C_18_H_18_O_7_	195.0916, 225.0763	[M+NH_4_]^+^	e
12	11.76	(2R)-5-(9H-pyrido [3,4-b]indol-1-yl)pentane-1,2,5-triol	287.1391	C_16_H_18_N_2_O_3_	286.1317	[M+H]^+^	g
13	11.87	(3R)-5,7-dihydroxy-3-(4′-hydroxybenzyl)-chroman-4-one	304.1144	C_16_H_14_O_5_	245.1856, 181.0501	[M+NH_4_]^+^	e
14	13.64	Isonarthogenin 3-O-β-D-glucopyranosyl-(1→4)-β-D-galactopyranoside	755.4151	C_39_H_62_O_14_	397.1967	[M+H]^+^	c
15	13.74	Rutin	628.7041	C_27_H_30_O_16_	611.1379, 465.1101	[M+NH_4_]^+^	e
16	13.88	(25S)-pratioside D_1_	915.4570	C_45_H_70_O_19_	453.3537	[M+H]^+^	c
17	13.95	Polygonatoside C	769.3996	C_39_H_60_O_15_	632.3469	[M+H]^+^	c
18	14.03	(25S)-spirost-5-en-3β,14α-diol 3-O-β-D-glucopyranosyl- (1→4)-β-D-galactopyranoside	755.4186	C_39_H_62_O_14_	350.2867, 173.1277	[M+H]^+^	c
19	14.08	Polygonatoside A	915.4570	C_45_H_70_O_19_	611.3829, 429.2907	[M+H]^+^	c
20	14.80	Pratioside D_1_	915.4518	C_45_H_70_O_19_	591.3515, 429.2989, 297.2206	[M+H]^+^	c
21	14.83	Pratioside C	1079.5345	C_51_H_82_O_24_	591.3515, 429.2989	[M+H]^+^	c
22	14.95	(25R)-furost-5-en-3β,22α,26-triol 26-O-β-D-glucopyranoside	617.3658	C_33_H_54_O_9_	427.2834, 120.0814	[M+Na] ^+^, [M+NH_4_]^+^	b
23	15.00	(25S)-kingianoside A	753.4026	C_39_H_60_O_14_	591.3515, 573.3401, 429.2989, 297.2206	[M+H]^+^	c
24	15.24	Diosgenin 3-O-α-L-rhamnopyranosyl (1→4)-β-D-glucopyranoside	740.4587	C_39_H_62_O_12_	322.2485	[M+NH_4_]^+^	c
25	15.55	Disporopsin	303.0871	C_16_H_14_O_6_	201.0517, 181.0501,	[M+H]^+^	e
26	15.60	Huangjinoside F	793.3937	C_39_H_62_O_15_	322.2487	[M+Na]^+^	c
27	15.87	(3β,22ξ,25R)-3-22-hydroxyfurost-5-en-26-yl-β-D-glucopyranoside	903.4886	C_45_H_74_O_18_	561.2966, 337.1713, 175.1193	[M+H]^+^	b
28	16.32	Huangjingenin	464.3313	C_27_H_42_O_5_	322.2481	[M+NH_4_]^+^	c
29	16.34	(3β,23S,25R)-23-hydroxy-12-oxospirost-5-en-3-yl-4-O-β-D-glucopyranosyl-β-D-galactopyranoside	786.4236	C_39_H_60_O_15_	387.1777, 147.0811	[M+NH_4_]^+^	c
30	17.20	Kingianoside B	737.4040	C_39_H_60_O_13_	453.3667, 211.0672	[M+H]^+^	c
31	17.37	(25S)-spirost-5-ene-3β-ol-3-O-β-D-galactopyranoside	577.3695	C_33_H_52_O_8_	253.1946	[M+H]^+^	c
32	17.38	Funkioside C	739.4225	C_39_H_62_O_13_	577.3708	[M+H]^+^	c
33	17.40	Polygonatosides A	577.3708	C_33_H_52_O_8_	415.3201, 253.1943	[M+H]^+^	c
34	18.09	Yamogenin 3-O-β-D-glucopyranosyl-(1→3)-[β-D-fucopyranosyl-(1→2)]-β-D-glucopyranosyl-(1→4)-β-D-galactopyranoside	1047.5289	C_51_H_82_O_22_	739.4229	[M+H]^+^	c
35	18.32	Kaempferol	287.0705	C_15_H_10_O_6_	258.0569, 168.0110	[M+H]^+^	e
36	22.46	(25R)-3β,14α-dihydroxy-spirost-5-ene	431.3141	C_27_H_42_O_4_	401.1942	[M+H]^+^	c
37	22.98	(6R,9R)-roseoside	823.4065	C_20_H_32_O_8_	279.1104	[2M+Na]^+^	g
38	23.27	Spiroakyroside	1092.5147	C_51_H_78_O_24_	591.3509	[M+Na]^+^, [M+NH_4_]^+^	c
39	23.50	Polygodoside A	1064.5237	C_50_H_78_O_23_	952.4227, 369.1949, 284.2569	[M+NH_4_]^+^	c
40	23.78	Gentrogenin	429.2991	C_27_H_40_O_4_	297.2206	[M+H]^+^	c
41	25.21	(3R)-5,7-dihydroxyl-6,8-dimethyl-3-(4′-hydroxylbenzyl)-chroman-4-one	315.1216	C_18_H_18_O_5_	209.0807,209.0814	[M+H]^+^	e
42	25.51	Polygonatone C	331.1176	C_18_H_18_O_6_	209.0807, 195.0657	[M+H]^+^	e
43	25.63	(20α,22R,25S)-Spirosta-5-ene-3β-ol	415.2259	C_27_H_42_O_3_	384.2042	[M+H]^+^	b
44	26.72	( ± )-5,7-dihydroxy-6,8-dimethyl-3-(2′-hydroxy-4′-methoxybenzyl)-chroman-4-one	345.1320	C_19_H_20_O_6_	272.2940, 209.0814	[M+H]^+^	e
45	27.07	Polygonatoside E′	1080.5530	C_51_H_82_O_23_	577.3644, 184.0728	[M+NH_4_]^+^	c
46	27.41	Diosgenin	415.3173	C_27_H_42_O_3_	279.2312, 397.3086	[M+H]^+^	c
47	27.42	Yamogenin 3-O-β-lycotetraoside	1055.4988	C_50_H_80_O_22_	659.2311, 437.1931	[M+Na]^+^	c
48	28.76	(3R)-5,7-dihydroxy-3-(2′-hydroxy-4′-methoxybenzyl)- chroman-4-one	317.1115	C_17_H_16_O_6_	290.6156, 181.0501	[M+H]^+^	e
49	31.97	Polygonatumoside C	1193.5678	C_56_H_88_O_27_	901.3682, 561.2792	[M+H]^+^	a
50	32.12	Yamogenin 3-O-β-D-glucopyranosyl-(1→4)-β-D-xylopyranoside	731.3993	C_38_H_60_O_12_	277.2160	[M+Na]^+^	c
51	32.94	(25S)-3-β-hydroxyspirost-5-en-12-one	429.2986	C_27_H_40_O_4_	285.1771, 327.2636	[M+H]^+^	c
52	33.23	(3R)-5,7-dihydroxyl-6-methyl-8-methoxyl-3-(4′-hydroxylbenzyl)-chroman-4-one	348.1500	C_18_H_18_O_6_	149.0236, 225.0763	[M+NH_4_]^+^	e
53	34.43	(3R)-5,7-dihydroxy-8-methyl-3-(4′-hydroxybenzyl)-chroman-4-one	301.1052	C_17_H_16_O_5_	285.0838, 195.0657	[M+H]^+^	e
54	35.54	(E)-3-(3′,4′-dihydroxybenzylidene)-5,7-dihydroxy-6,8-dimethylchroman-4-one	329.1692	C_18_H_16_O_6_	273.1056, 209.0814	[M+H]^+^	e
55	37.54	(3S)-3,5,7-trihydroxy-6-methyl-3-(4′-methoxybenzyl) chroman-4-one	331.1166	C_18_H_18_O_6_	223.0606, 211.0606, 167.0344	[M+H]^+^	e
56	37.89	Linoleic acid	263.2373	C_18_H_32_O_2_	207.1087	[M+H-H_2_O]^+^	g
57	39.41	Dioscin	869.4951	C_45_H_72_O_16_	760.5807, 321.3148	[M+H]^+^	c
58	39.8	Huangjinoside I	774.4222	C_38_H_60_O_15_	184.0739	[M+NH_4_]^+^	c
59	39.95	9,19-cyclolart-25-en-3,24 (R)-diol	481.3506	C_30_H_50_O_2_	227.1273	[M+K]^+^	d
60	40.32	Funkioside B	631.3796	C_34_H_56_O_9_	507.3658	[M+Na]^+^	b
61	40.34	(E)-8,11,12-trihydroxyoctadec-9-enoic acid	295.2262	C_18_H_34_O_5_	122.1032	[M+H-2H_2_O]^+^	g
62	1.16	D-fructose	341.2071	C_6_H_12_O_6_	160.9872, 143.0502, 119.0528, 89.0275	[2M-H-H_2_O]^−^	f
63	1.22	L-Pyroglutamic acid	128.0348	C_5_H_7_NO_3_	96.9626	[M-H]^−^	g
64	2.37	Saccharide residues	191.0302	C_10_H_7_O_4_	111.0501, 191.0245	[M-H]^−^	f
65	3.59	Fructose or glucosyl residues	323.1146	C_12_H_20_O_10_	161.0547, 143.0428	[2M-2H_2_O-H]^−^	f
66	4.02	Fructose or glucosyl residues	323.1146	C_12_H_20_O_10_	161.0547, 89.0295	[2M-2H_2_O-H]^−^	f
67	5.31	Sucrose	341.1202	C_12_H_22_O_11_	179.0613	[M-H]^−^	f
68	6.26	1-Kestose	503.1743	C_18_H_32_O_16_	341.1201, 179.0613	[M-H]^−^	f
69	6.47	Raffinose	503.1743	C_18_H_32_O_16_	341.1239, 323.1072	[M-H]^−^	f
70	7.23	Nistose	665.2316	C_24_H_42_O_21_	485.1704, 341.1201	[M-H]^−^	f
71	7.54	Inulin-type tetrasaccharide	665.2316	C_24_H_42_O_21_	485.1704, 341.1201	[M-H]^−^	f
72	7.90	Inulin-type pentasaccharide	827.2936	C_30_H_52_O_26_	545.1896, 341.1201	[M-H]^−^	f
73	8.11	Inulin-type pentasaccharide	827.2936	C_30_H_52_O_26_	341.1201, 179.0613	[M-H]^−^	f
74	8.66	Inulin-type hexasaccharide	989.3560	C_36_H_62_O_31_	179.0613	[M-H]^−^	f
75	9.18	Inulin-type heptasaccharide	1151.4080	C_42_H_72_O_36_	989.3560	[M-H]^−^	f
76	9.73	Inulin-type octasaccharide	1313.4729	C_48_H_82_O_41_	1151.4149, 656.2355	[M-H]^−^	f
77	10.07	Inulin-type nine glycans	1475.5381	C_54_H_92_O_46_	1313.4729, 737.2625	[M-H]^−^	f
78	10.44	Inulin-type ten glycans	1637.5312	C_60_H_102_O_51_	818.2943, 737.2625	[M-H]^−^	f
79	10.79	Inulin-type eleven glycans	1799.5812	C_66_H_112_O_56_	899.3264, 818.2943	[M-H]^−^	f
80	11.08	Inulin-type twelve glycans	1961.6432	C_72_H_122_O_61_	980.3521, 899.3264	[M-H]^−^	f
81	11.35	Inulin-type thirteen glycans	2123.6914	C_78_H_132_O_66_	1061.3872, 980.3585	[M-H]^−^	f
82	11.57	Inulin-type fourteen glycans	2285.7421	C_84_H_142_O_71_	1142.9181, 1061.3872	[M-H]^−^	f
83	11.78	Inulin-type fifteen glycans	2447.8032	C_90_H_152_O_76_	1223.9530, 1142.4196	[M-H]^−^	f

Note: C. denotes classification; a: cholestane saponins; b: furostanol saponins; c: spirostanol saponins; d: triterpenoid saponins; e: flavonoids; f: oligosaccharides or monosaccharides; g: other.

## Data Availability

The data presented in this study are available in Supplementary Material.

## Supplementary Materials

The following supporting information can be downloaded at: https://www.mdpi.com/article/10.3390/molecules27185869/s1, Figure S1: Base peak intensity (BPI) chromatogram of secondary metabolites in raw Polygonatum cyrtonema rhizome in positive (A) and negative ion mode (B); Table S1: Method validation of UPLC-Q-TOF-MS/MS of secondary metabolites; Table S2: Method validation of UPLC-Q-TOF-MS/MS of monosaccharides and oligosaccharides; Table S3: Calibration curves, sensitivity, precision, repeatability, and stability of the monosaccharide composition determination assay; Table S4: Calibration curve, sensitivity, precision, repeatability, and stability of the molecular weight determination assay; Table S5: Calibration curves and sensitivity of the glycoprotein, uronic acid, and total polysaccharide determination assays. Table S6: Summary of 38 batches of PCR samples.

## Sample Availability

Samples of the compounds are not available from the authors.

## Abbreviations

PCR, Polygonatum cyrtonema rhizome; PCA, principal component analysis; PLS-DA, partial least-squares discriminant analysis; VIP, variable importance parameters; PCP, Polygonatum cyrtonema polysaccharides; UPLC-Q-TOF-MS/MS, ultra-high-performance liquid chromatography tandem quadrupole time-of-flight mass spectrometry; HPLC-PDA, high-performance liquid chromatography-photo diode array; HPGPC-RID, high-performance gel permeation chromatography-refractive index detector; PMP, 1-phenyl-3-methyl-5-pyrazolone; FOS, fructo-oligosaccharides; DP, degree of polymerization; TCM, traditional Chinese medicine.

## References

[1] Chinese Pharmacopoeia Commission (2020). Pharmacopoeia of the People’s Republic of China.

[2] Yan H.L., Lu J.M., Wang Y.F., Gu W., Yang X.X., Yu J. (2017). Intake of total saponins and polysaccharides from *Polygonatum kingianum* affects the gut microbiota in diabetic rats. Phytomedicine.

[3] Lu J.M., Wang Y.F., Yan H.L., Lin P., Gu W., Yu J. (2016). Antidiabetic effect of total saponins from *Polygonatum kingianum* in streptozotocin-induced diabetic rats. J. Ethnopharmacol..

[4] Guo C., Li C., Yu Y., Chen W., Ma T., Zhou Z. (2016). Antihyperglycemic and antihyperlipidemic activities of protodioscin in a high-fat diet and streptozotocin-induced diabetic rats. RSC Adv..

[5] Du L., Nong M.N., Zhao J.M., Peng X.M., Zong S.H., Zeng G.F. (2016). *Polygonatum sibiricum* polysaccharide inhibits osteoporosis by promoting osteoblast formation and blocking osteoclastogenesis through Wnt/β-catenin signalling pathway. Sci. Rep..

[6] Zhao P., Zhao C.C., Li X., Gao Q.Z., Huang L.Q., Xiao P.G. (2018). The genus *Polygonatum*: A review of ethnopharmacology, phytochemistry and pharmacology. J. Ethnopharmacol..

[7] Zhang G.X., Zeng X.C., Zhang R., Liu J., Zhang W.C., Zhao Y.J., Zhang X.Y., Wu Z.X., Tan Y.H., Wu Y.Y. (2016). Dioscin suppresses hepatocellular carcinoma tumor growth by inducing apoptosis and regulation of TP53, BAX, BCL2 and cleaved CASP3. Phytomedicine.

[8] Zhao S., Niu F., Xu C.Y., Liu Y., Ye L., Bi G.B., Chen L., Tian G., Nie T.H. (2016). Diosgenin prevents bone loss on retinoic acid-induced osteoporosis in rats. Ir. J. Med. Sci..

[9] Zhao X.Y., Li J. (2014). Chemical constituents of the genus *Polygonatum* and their role in medicinal treatment. Nat. Prod. Commun..

[10] Zhang H., Yang F., Jin Q., Song X.C., Hu Z.F., Zhu D.N., Yu B.Y. (2010). Homoisoflavonoids from the fibrous roots of *Polygonatum odoratum* with glucose uptake-stimulatory activity in 3T3-L1 adipocytes. J. Nat. Prod..

[11] Liu F., Liu Y.H., Meng Y.W., Yang M., He K. (2004). Structure of polysaccharide from *Polygonatum cyrtonema* Hua and the antiherpetic activity of its hydrolyzed fragments. Antivir. Res..

[12] He L.L., Yan B.X., Yao C.Y., Chen X.Y., Li L.W., Wu Y.J., Song Z.J., Song S.S., Zhang Z.F., Luo P. (2020). Oligosaccharides from *Polygonatum Cyrtonema* Hua: Structural characterization and treatment of LPS-induced peritonitis in mice. Carbohyd. Polym..

[13] Zhao P., Li X., Wang Y., Yan L., Guo L., Huang L., Gao W. (2020). Characterisation and saccharide mapping of polysaccharides from four common Polygonatum spp.. Carbohyd. Polym..

[14] Yelithao K., Surayot U., Lee J.H., You S. (2016). Raw264.7 cell activating glucomannans extracted from rhizoma of Polygonatum sibiricum. Prev. Nutr. Food Sci..

[15] Zeng G.F., Zhang Z.Y., Lu L., Xiao D.Q., Xiong C.X., Zhao Y.X., Zong S.H. (2011). Protective effects of *Polygonatum sibiricum* polysaccharide on ovariectomy-induced bone loss in rats. J. Ethnopharmacol..

[16] Wang Y., Qin S.C., Pen G.Q., Chen D., Han C., Miao C.R., Lu B.J., Su C., Feng S.L., Li W. (2017). Potential ocular protection and dynamic observation of *Polygonatum sibiricum* polysaccharide against streptozocin-induced diabetic rats’ model. Exp. Biol. Med..

[17] Wang J., Yue Y.D., Tang F., Tao W.Q. (2011). Comparative analysis of volatile fractions in Polygonati Rhizoma and its processed products by GC-MS. China J. Chin. Mater. Med..

[18] Jin J., Lao J., Zhou R.R., He W., Qin Y., Zhong C., Xie J., Liu H., Wan D., Zhang S.H. (2018). Simultaneous identification and dynamic analysis of saccharides during steam processing of rhizomas of Polygonatum cyrtonema by HPLC-QTOF-MS/MS. Molecules.

[19] Sun T.T., Zhang H., Li Y., Liu Y., Dai W., Fang J., Cao C., Die Y., Liu Q., Wang C.L. (2019). Physicochemical properties and immunological activities of polysaccharides from both crude and wine-processed *Polygonatum sibiricum*. Int. J. Biol. Macromol..

[20] Wu W.J., Huang N.W., Huang J.P., Wang L.L., Wu L.L., Wang Q., Zhao H.J. (2021). Effects of the steaming process on the structural properties and immunological activities of polysaccharides from Polygonatum cyrtonema. J. Funct. Foods.

[21] Zhou Q., Wu P., Zhang X.L., Zhang Y.X., Zhang Z.H., Wang Y. (2021). Effects of *Polygonatum cyrtonema* and braising of *Polygonatum cyrtonema* on gastrointestinal motility and hormonal readiness in spleen-deficiency mouse. Lishizhen Med. Mater. Med. Res..

[22] Fiehn O., Kopka J., Dormann P., Altmann T., Trethewey R.N., Willmitzer L. (2001). Metabolite profiling for plant functional genomics. Nat. Biotechnol..

[23] Hu C.X., Xu G.W. (2014). Metabolomics and traditional Chinese medicine. Trend. Anal. Chem..

[24] Zhang A., Sun H., Wang Z.G., Sun W.J., Wang P., Wang X.J. (2010). Metabolomics: Towards understanding traditional Chinese medicine. Planta Med..

[25] Hu D.J., Cheong K.L., Zhao J., Li S.P. (2013). Chromatography in characterization of polysaccharides from medicinal plants and fungi. J. Sep. Sci..

[26] Hart G.W., Copeland R.J. (2010). Glycomics hits the big time. Cell.

[27] Wang Y., Zhang J.J., Wang Z.X., Cui F., Zhang Q.N., Song P.P., Li B., Tang Z.S., Hu F.D., Shi X.F. (2022). Characterization of chemical composition variations in raw and processed Codonopsis Radix by integrating metabolomics and glycomics based on multiple chromatography-mass spectrometry technology. J. Sep. Sci..

[28] Zhou L., Xu J.D., Zhou S.S., Mao Q., Kong M., Shen H., Li X.Y., Duan S.M., Xu J., Li S.L. (2016). Integrating targeted glycomics and untargeted metabolomics to investigate the processing chemistry of herbal medicines, a case study on Rehmanniae Radix. J. Chromatogr. A.

[29] Yip K.M., Xu J., Zhou S.S., Lau Y.M., Chen Q.L., Tang Y.C., Yang Z.J., Yao Z.P., Ding P., Chen H.B. (2019). Characterization of chemical component variations in different growth years and tissues of Morindae Officinalis Radix by integrating metabolomics and glycomics. J. Agric. Food Chem..

[30] Yu X.A., Ge A.H., Zhang L., Li J., An M.R., Cao J., He J., Gao X.M., Chang Y.X. (2017). Influence of different processing times on the quality of Polygoni Multiflora Radix by metabolomics based on ultra high performance liquid chromatography with quadrupole time-of-flight mass spectrometry. J. Sep. Sci..

[31] Xue S.J., Wang L.L., Chen S.Q., Cheng Y.X. (2018). Simultaneous analysis of saccharides between fresh and processed Radix Rehmanniae by HPLC and UHPLC-LTQ-Orbitrap-MS with multivariate statistical analysis. Molecules.

[32] Liu Z.R., Lou Z.Y., Ding X., Li X., Qi Y.P., Zhu Z.Y., Chai Y.F. (2013). Global characterization of neutral saccharides in crude and processed Radix Rehmanniae by hydrophilic interaction liquid chromatography tandem electrospray ionization time-of-flight mass spectrometry. Food Chem..

[33] Ahn M.J., Kim J. (2005). Identification and quantification of steroidal saponins in Polygonatum species by HPLC/ESI/MS. Arch. Pharmacal. Res..

[34] Tomoda M., Satoh N., Sugiyama A. (1973). Isolation and characterization of fructans from *Polygonatum odoratum* var. japonicum rhizomas. Chem. Pharm. Bull..

[35] Tang L.Y., Wu H.W., Zhou X.D., Xu Y.L., Zhou G.H., Wang T., Kou Z.Z., Wang Z.J. (2015). Discrimination of Semen cassia from two related species based on the multivariate analysis of high-performance liquid chromatography fingerprints. J. Sep. Sci..

[36] Zhang D.K., Han X., Li R.Y., Niu M., Dong Q., Yang M., Wang J.B., Xiao X.H. (2016). Investigation of the chemical markers for experiential quality evaluation of crudeaconite by UHPLC-Q-TOF-MS. J. Sep. Sci..

[37] Hotelling H. (1933). Analysis of a complex of statistical variables into principal components. J. Educ. Psychol..

[38] Perez-Enciso M., Tenenhaus M. (2003). Prediction of clinical outcome with microarray data: A partial least squares discriminant analysis (PLS-DA) approach. Hum. Genet..

[39] Rosatella A.A., Simeonov S.P., Frade R.F.M., Afonso C.A.M. (2011). 5-hydroxymethylfurfural (HMF) as a building block platform: Biological properties, synthesis and synthetic applications. Green Chem..

[40] Georgelis N., Fencil K., Richael C.M. (2018). Validation of a rapid and sensitive HPLC/MS method for measuring sucrose, fructose and glucose in plant tissues. Food Chem..

[41] Liu S.C., Yang D.J., Jin S.Y., Hsu C.H., Chen S.L. (2008). Kinetics of color development, pH decreasing, and anti-oxidative activity reduction of Maillard reaction ingalactose/glycine model systems. Food Chem..

[42] Chen X.Q., Ye Y., Cheng H., Jiang Y.W., Wu Y.L. (2009). Thermal effects on the stability and antioxidant activity of an acid polysaccharide conjugate derived from green tea. J. Agric. Food Chem..

[43] Melton L.D., Mindt L., Rees D.A., Sanderson G.R. (1976). Covalent structure of the extracellular polysaccharide from Xanthomonas campestris: Evidence from partial hydrolysis studies. Carbohydr. Res..

[44] Lan G.S., Chen H.X., Chen S.H., Tian J.G. (2012). Chemical composition and physicochemical properties of dietary fiber from *Polygonatum odoratum* as affected by different processing methods. Food Res. Int..

[45] Pierce J., Suelter C.H. (1977). An evaluation of the Coomassie brilliant blue G-250 dye-binding method for quantitative protein determination. Anal. Biochem..

[46] Selvendran R.R., March J.F., Ring S.G. (1979). Determination of aldoses and uranic acid content of vegetable fiber. Anal. Biochem..

