# Supplementary material for: Research on Processing-Induced Chemical Variations in Polygonatum Cyrtonema Rhizome by Integrating Metabolomics and Glycomics
Source: Molecules. 2022 Sep 10;27(18):5869. doi: 10.3390/molecules27185869 (PMC9506285; doi:10.3390/molecules27185869)
Supplement: Supplementary file 1 [file molecules-27-05869-s001.zip › molecules-1880185-supplementary.pdf]

Supplementary File

Research on Processing-Induced Chemical Variations in Polygonatum Cyrtonema Rhizome by Integrating Metabolomics and Glycomics

Tong Jiang [1], Tong Wu [1], Peiyun Gao [2], Lixia Wang [1], Xiaoyun Yang [1], Xiaoxu Chen [1], Yingying Chen [1], Chunyu Yue [2], Keqing Liang [1], Liying Tang [1,*] and Zhuju Wang [1,*]

[1] Institute of Chinese Materia Medica, China Academy of Chinese Medical Science, Beijing 100700, China

[2] College of Pharmacy, Henan University of Chinese Medicine, Zhengzhou 450046, China

* Correspondence: lytang@icmm.ac.cn (L.T.); zjwang@icmm.ac.cn (Z.W.)

Section 1. Method optimization and validation

1.1. Method optimization and validation of secondary metabolites

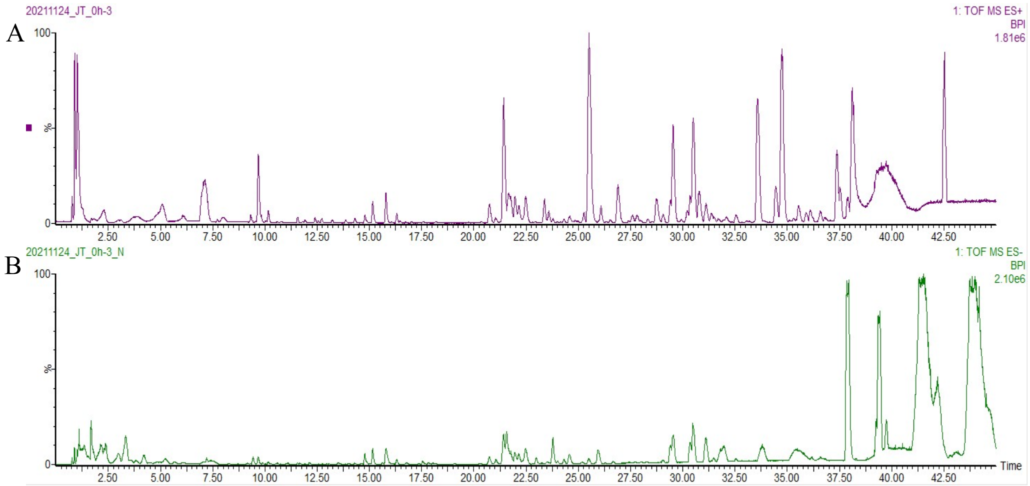

Figure. S1 The base peak intensity (BPI) chromatogram of secondary metabolites in raw Polygonatum cyrtonema rhizome in positive (A) and negative ion mode (B).

More secondary metabolites were detected in positive ion mode, so the analysis of secondary metabolites was performed in positive ion mode (Fig. S1).

A mixed standard solution was prepared with methanol at different concentrations which contained 0.89 mg/mL of kaempferol, 0.64 mg/mL of rutin, 0.52 mg/mL of (20α,22R,25S)-Spirosta-5-ene-3β-ol. The method validation included precision,

repeatability and stability. The mixed standard solutions were used for method validation. The precision was investigated by one sample with six replicate injections. The repeatability of the method was assessed by performing six replicate solutions. The stability of those analytes was assessed by analyzing the solution at 0, 2, 4, 6, 8, 12 and 24 h. The validation was expressed as the RSD and the RSD values were less than 3.0 % (Table S1). The above results showed that UPLC-Q-TOF-MS/MS method could be used for the analysis of PCR metabolites.

Table S1 The method validation of UPLC-Q-TOF-MS/MS of secondary metabolites

Compound	Precision (RSD%)	Repeatability (RSD%)	Stability (RSD%)
kaempferol	1.91	1.69	1.64
rutin	3.00	2.85	2.43
(20α,22R,25S)-Spirosta-5-ene-3β-ol	2.87	2.76	2.77

1.2. Method validation of monosaccharides and oligosaccharides

A mixed sugar standard solution was prepared with 20% acetonitrile at different concentrations which contained 1.048 mg/mL of D-fructose, 1.028 mg/mL of sucrose, 1.01 mg/mL of 1-kestose, and 1.05 mg/mL of nistose. The precision, stability and repeatability of the method were verified by the mixed standard of sugar and the RSD values were less than 3.0 %. The above results showed that UPLC-Q-TOF-MS/MS method could be used for the analysis of monosaccharides and oligosaccharides in PCR.

Table S2 The method validation of UPLC-Q-TOF-MS/MS of monosaccharides and oligosaccharide

Compound	Precision (RSD%)	Repeatability (RSD%)	Stability (RSD%)
D-fructose	2.81	3.00	2.65
sucrose	1.69	1.75	1.63
1-kestose	2.75	2.66	2.97
nistose.	2.34	2.49	2.14

1.3. Method validation of the monosaccharide composition determination.

The precision, stability and repeatability of the method were verified by the sample solution and the RSD values were less than 3.0 %.

Table S3 Calibration curves, sensitivity, precision, repeatability, and stability of the monosaccharide composition determination assay

Compound	Range (μg)	Equation	R^2	Precision (RSD%)	Repeatability (RSD%)	Stability (RSD%)
Man	10-500	y=29770x+27170.3	0.9999	0.07	1.17	0.86
Rib	5-250	y=42127.2x-129706.4	0.9999	1.51	0.49	1.00
GlcA	5-500	y=23496.7x+75784.2	0.9999	-	-	-
GalA	5-500	y=25336.6x+27998.5	0.9999	1.55	2.21	1.33
Fru	5-500	y=21670x+134629.2	0.9999	0.23	0.21	0.31
Gal	5-500	y=27127.6x+104091.9	0.9999	0.21	0.22	0.24
Xyl	5-500	y=35112x-2450.3	0.9999	0.92	1.90	1.78

1.4. Method validation of the molecular weight determination.

Table S4 Calibration curve, sensitivity, precision, repeatability, and stability of the molecular weight determination assay

Equation	Range (Da)	R^2	Precision (RSD%)	Repeatability (RSD%)	Stability (RSD%)
$lgM_w = -0.7229\ t + 9.3918$	180-300600	0.9959	0.6	0.59	2.99

1.5. Calibration curves and sensitivity of the glycoprotein, uronic acid and total polysaccharide determination assays.

Table S5 Calibration curves and sensitivity of the glycoprotein, uronic acid and total polysaccharide determination assays

Detection components	Equation	Range	R^2
glycoprotein	y=4.3178x+0.0883	0.0329-0.1975 mg	0.9995
uronic acid	y=3.623x+0.0208	0.0208-0.1768 mg	0.9994
total polysaccharide	y=0.0045x+0.0067	0-60 ug	0.9998

Section 2. The collection information of plant material is listed as follows.

Table S6 Summary of information of 38 batches of PCR samples.

Sample No.	Harvesting time	planting place/time	Processing time
S1	30 October 2020	Standardized planting base, Yongzhou, Hunan/2017	Raw
S2	30 October 2020	Standardized planting base, Yongzhou, Hunan/2017	Raw
S3	30 October 2020	Standardized planting base, Yongzhou, Hunan/2017	Raw

Sample No.	Harvesting time	planting place/time	Processing time
S4	30 October 2020	Standardized planting base, Yongzhou, Hunan/2017	Raw
S5	30 October 2020	Standardized planting base, Yongzhou, Hunan/2017	Raw
S6	-	Processing in laboratory	1h
S7	-	Processing in laboratory	1h
S8	-	Processing in laboratory	1h
S9	-	Processing in laboratory	2h
S10	-	Processing in laboratory	2h
S11	-	Processing in laboratory	2h
S12	-	Processing in laboratory	3h
S13	-	Processing in laboratory	3h
S14	-	Processing in laboratory	3h
S15	-	Processing in laboratory	4h
S16	-	Processing in laboratory	4h
S17	-	Processing in laboratory	4h
S18	-	Processing in laboratory	6h
S19	-	Processing in laboratory	6h
S20	-	Processing in laboratory	6h
S21	-	Processing in laboratory	8h
S22	-	Processing in laboratory	8h
S23	-	Processing in laboratory	8h
S24	-	Processing in laboratory	10h
S25	-	Processing in laboratory	10h
S26	-	Processing in laboratory	10h
S27	-	Processing in laboratory	12h
S28	-	Processing in laboratory	12h
S29	-	Processing in laboratory	12h
S30	-	Processing in laboratory	14h
S31	-	Processing in laboratory	14h
S32	-	Processing in laboratory	14h
S33	-	Processing in laboratory	16h
S34	-	Processing in laboratory	16h
S35	-	Processing in laboratory	16h
S36	-	Processing in laboratory	18h
S37	-	Processing in laboratory	18h
S38	-	Processing in laboratory	18h
